# Breast Metastasis From Cutaneous Melanoma: Beyond the Infiltrating Ductal Carcinoma

**DOI:** 10.7759/cureus.60931

**Published:** 2024-05-23

**Authors:** Marisol Ramos Portales, Carmen G Bermúdez Barrientos, Nishdaly A Rodríguez Valencia, Brizio Moreno Jaime

**Affiliations:** 1 General Surgery, Institute for Social Security and Services for State Workers Regional Hospital, Leon, MEX; 2 Medical Oncology, Institute for Social Security and Services for State Workers Regional Hospital, Leon, MEX; 3 Dermatology, Institute for Social Security and Services for State Workers Regional Hospital "Lic. Adolfo Lopez Mateos", Mexico City, MEX

**Keywords:** neoplasm of uncertain behaviour, cutaneous malignant melanoma, malignant melanoma metastasis, breast cancer, ­skin cancer

## Abstract

Breast metastases of extramammary origin are an extremely rare entity. Solid organ metastases to the breast include malignant melanoma, epithelial carcinoma (adenocarcinoma and squamous cell carcinoma of the lung and gastrointestinal tract), and sarcoma. A breast neoplasm can be caused by a primary tumor, in-transit metastasis, breast metastasis, and skin metastasis. A 42-year-old female patient presented with a hyperpigmented lesion on the first finger of her left hand. An incisional biopsy was carried out, reporting pigmented epithelioid melanoma. Amputation of the finger was performed, as well as an axillary sentinel lymph node excision. Later during the treatment and follow-up by medical oncology, a breast tumor was located, followed by a protocol and the approach of possible differential diagnoses. Finally, it was characterized as metastatic cutaneous melanoma. The therapeutic approach regarding the possible origin of the metastatic neoplastic character of breast tumors culminated in this case in the palliative treatment with immunotherapy of cutaneous malignant melanoma. The diagnosis of breast metastases from cutaneous malignant melanoma is a real challenge, so an extensive history and high clinical suspicion are crucial in order to provide adequate treatment, despite the gloomy prognosis.

## Introduction

Breast metastases of extramammary origin are an extremely rare entity and rates range from 0.5% to 2% of all breast neoplasms. The first case of extramammary breast metastases was reported in 1903 [1]. Most of these represent metastases from carcinomas of the contralateral breast. Hematologic neoplasms represent 1% of all malignant breast tumors. Solid organ metastases account for less than 1% of all breast neoplasms, including melanoma, carcinoma (adenocarcinoma and squamous cell carcinoma of the lung and gastrointestinal tract, renal and prostate adenocarcinoma), carcinoid tumor, and sarcoma [1-3]. Metastasis presenting as a breast tumor has been reported to constitute 1.3-6.6% of all breast tumors.

A breast neoplasm can be caused by a primary tumor, in-transit metastasis, breast metastasis, and skin metastasis [2]. Melanoma metastasis to the breast often presents as a mass in the upper outer quadrant. This presentation is usually more common in young women. Breast metastases are more difficult to diagnose than primary breast cancers based solely on imaging tests. Microcalcifications are a rare finding in cancers that metastasize to the breast [2]. 

Melanoma is the leading cause of death from skin cancer and up to 90% of cases are extremely aggressive, have an unpredictable evolution and a low survival rate, metastasize in 20% of cases, and spread throughout via hematogenous or lymphatic [1], commonly to the lungs, brain, liver or any other solid organ. It is worth mentioning that less than 5% of melanoma metastasis corresponds to the breast [4].

It is important to carry out an adequate anamnesis and recognize breast metastasis as a potential differential diagnosis for a breast neoplasm [4]. Indeed, it is crucial to plan an appropriate treatment since melanoma is the second most aggressive skin cancer after Merkel cell carcinoma with a five-year survival rate of 5-19% [1].

## Case presentation

A 42-year-old female patient, in October 2018, presented with a hyperpigmented lesion in the volar surface of the distal phalanx of the first finger of her left hand, first noticed three months ago, which progressively increased in size, until reaching 1 cm in its major axis. Personal medical records report hypothyroidism of half a year of evolution in treatment with levothyroxine, gynecologic and obstetric history of four gestations, one cesarean section in 2007, two deliveries in 2009 and 2014, one miscarriage, onychomycosis in the first finger of the left hand in 2014, managed successfully with azole antifungal treatment, cervical-vaginal cytology with low-grade squamous intraepithelial lesion, and mild dysplasia treated successfully with loop electrosurgical excision procedure. In addition, the oncological family history reveals paternal grandmother had stomach cancer, a paternal aunt had pancreatic cancer, and a maternal aunt had uterine cancer.

In October 2018, after her presentation, an incisional biopsy of the first finger of her left hand was performed. The histopathological report showed tissue fragment of 0.5x0.3x0.2 cm and skin with dense stratum corneum, which showed intact epidermis and presence throughout the dermis evaluated of infiltration by epithelioid malignant neoplasm of malignant pigmented melanocytes; this lesion occupied the total thickness of the biopsy (0.2 mm). The diagnostic impression was non-ulcerated pigmented epithelioid melanoma. The mitotic index was 3 mitoses/mm^2^, Breslow greater than 2 mm (sample thickness), and minimum Clark level of 2 (papillary dermis).

It was decided to perform an amputation of the first finger of the left hand in November 2018 because two-thirds of the thickness of the finger was affected. In addition, a sentinel lymph node biopsy using a single dye technique with patent blue was performed, and one axillary lymph node was excised. The definitive histopathological result, reported in December 2018, stated that the piece had dimensions of 5.2 cm in the major axis and 1.0 cm in major diameter, and identified a tip-pigmented lesion measuring 1.0 x 0.9 cm along major axes and 1.0 cm deep with subungual extension, without contact with bone. The sentinel lymph node was 0.7 x 0.6 cm. Diagnostic impression was pigmented epithelioid melanoma infiltrating papillary and deep dermis with horizontal and vertical growth pattern, mild lymphoid infiltrate, without perineural or lymphatic infiltration, free borders of 3 cm, without bone involvement, Breslow 10 mm, and Clark level IV. No mutations of the BRAF genotype were detected. Sentinel node biopsy was negative for malignancy. Subsequently, the patient remained only under surveillance without any additional treatment.

**Figure 1:**
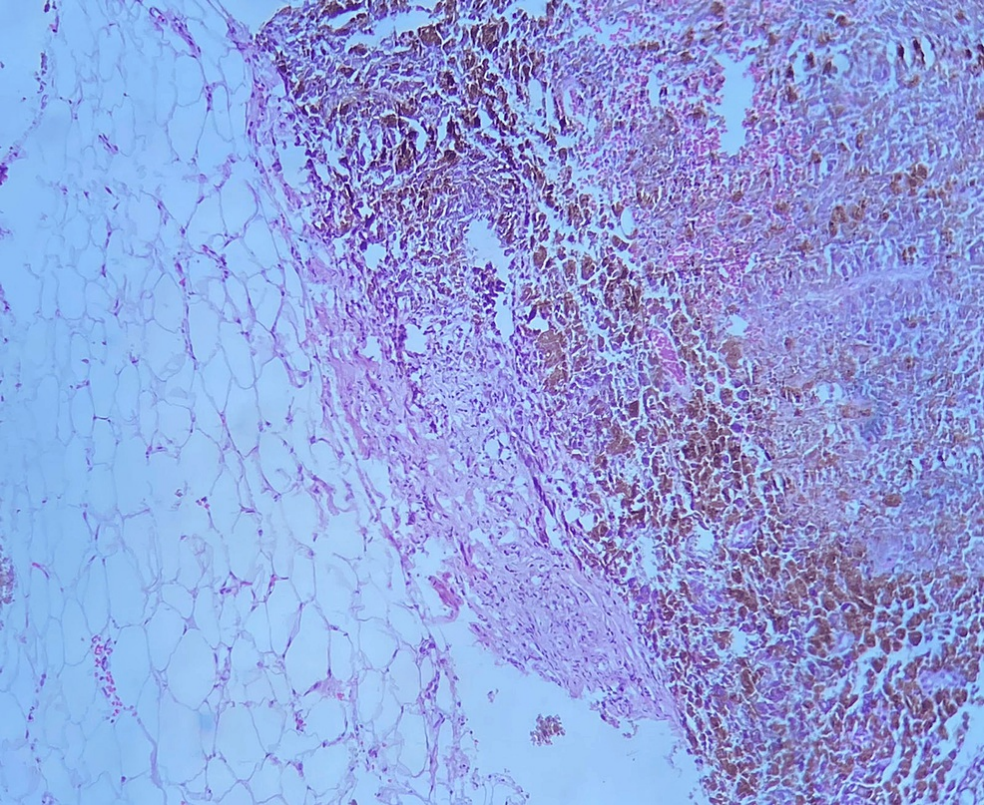
TruCut biopsy of the left breast nodule. In this section, breast parenchyma is observed, along with adjacent adipose tissue, infiltrated by a malignant neoplasm constituted by melanoma metastasis.

**Figure 2:**
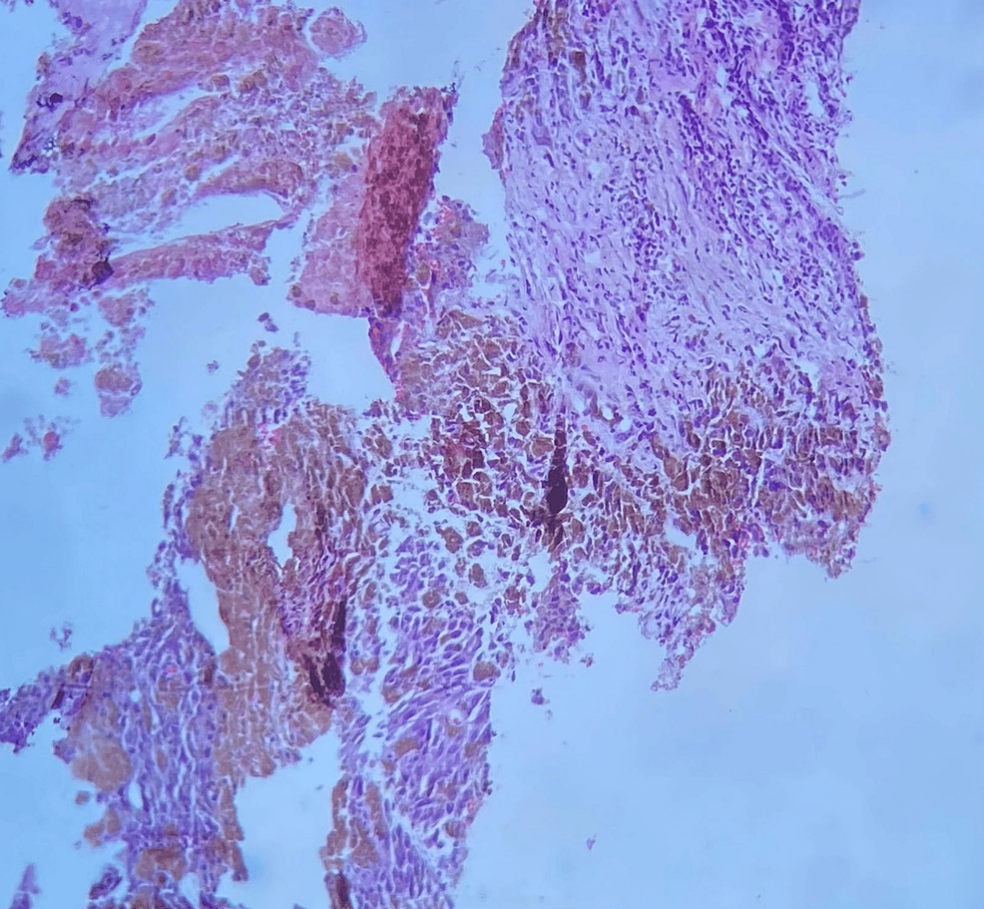
Histology of melanoma metastasis to the breast. A malignant neoplasm is discerned formed by cells with hyperpigmented cytoplasm and nuclei with the presence of a prominent nucleolus; these cells are arranged in irregular nests.

During a follow-up in June 2021, an elevation of lactate dehydrogenase of 643 U/l was noted and metastatic lesions were identified in a chest X-ray as well as liver ultrasound, which was corroborated with a computed tomography (CT) scan. It is worth mentioning that during the physical examination, a 4 cm painless nodule in the left breast parenchyma was found, as well as a 1.5 cm lymph node in the left hemi-neck, level V. Breast ultrasound was performed in June 2021, and in the lower quadrant of the left breast, several nodules were identified forming a conglomerate; the largest measured 26x14x20 mm and showed peripheral vascularity and rigid behavior on elastography. Other smaller satellite nodules measured 11, 7, 6, and 8 mm. Left axillary adenopathy was seen with loss of morphology. Additionally, adenopathy was identified in the left side of the neck, region V of 17x14 mm, besides and indurated and below the pectoral muscle. At the level of the third left rib, a lesion with similar characteristics was observed, concluding a Breast Imaging-Reporting and Data System (BI-RADS) 4C. Breast and right axillary region were of normal characteristics. It was decided to perform a TruCut biopsy of the left breast guided by ultrasound showed a presence of a large lesion in the left lobe in the segments III and IVA and confirmed the diagnosis of malignant melanoma (Figures 1-2).

**Figure 3:**
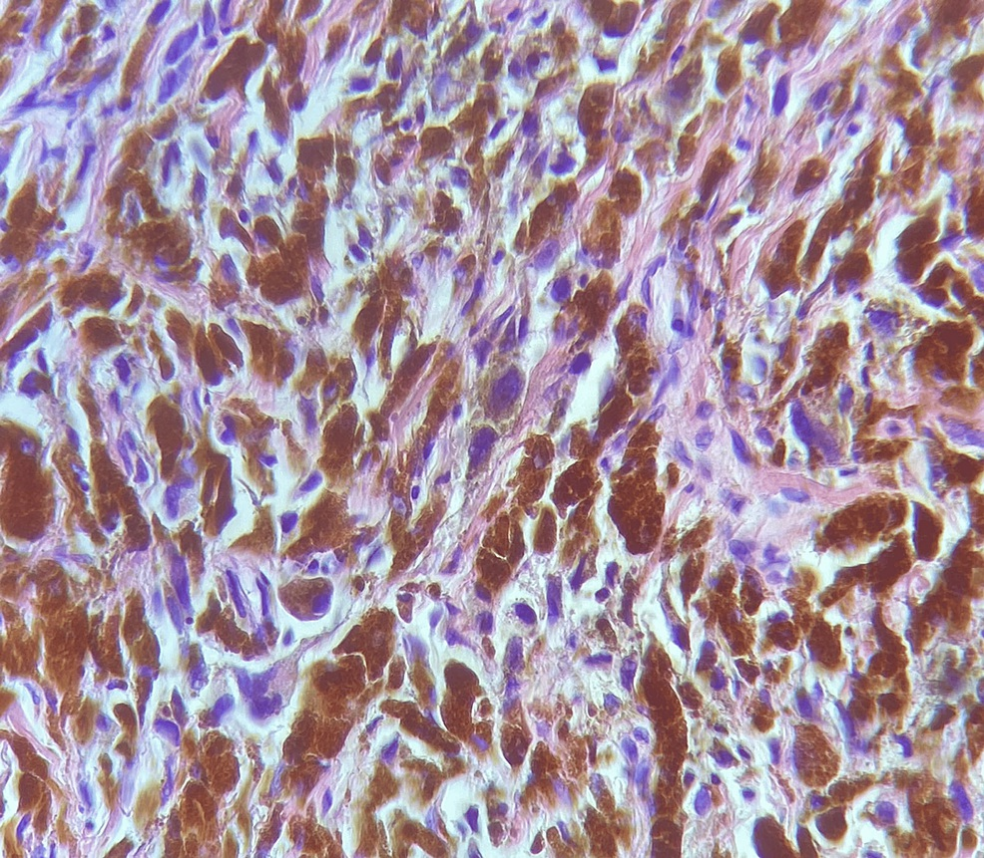
TruCut biopsy of the left axillary lymph node. In this section, residual lymphoid tissue is observed, as well as a malignant neoplasm consisting of cells with hyperpigmented cytoplasm and nuclei with prominent nucleoli.

TruCut biopsy of the left axillary lymph node confirmed the diagnosis of metastatic malignant melanoma (Figure 3).

**Figure 4:**
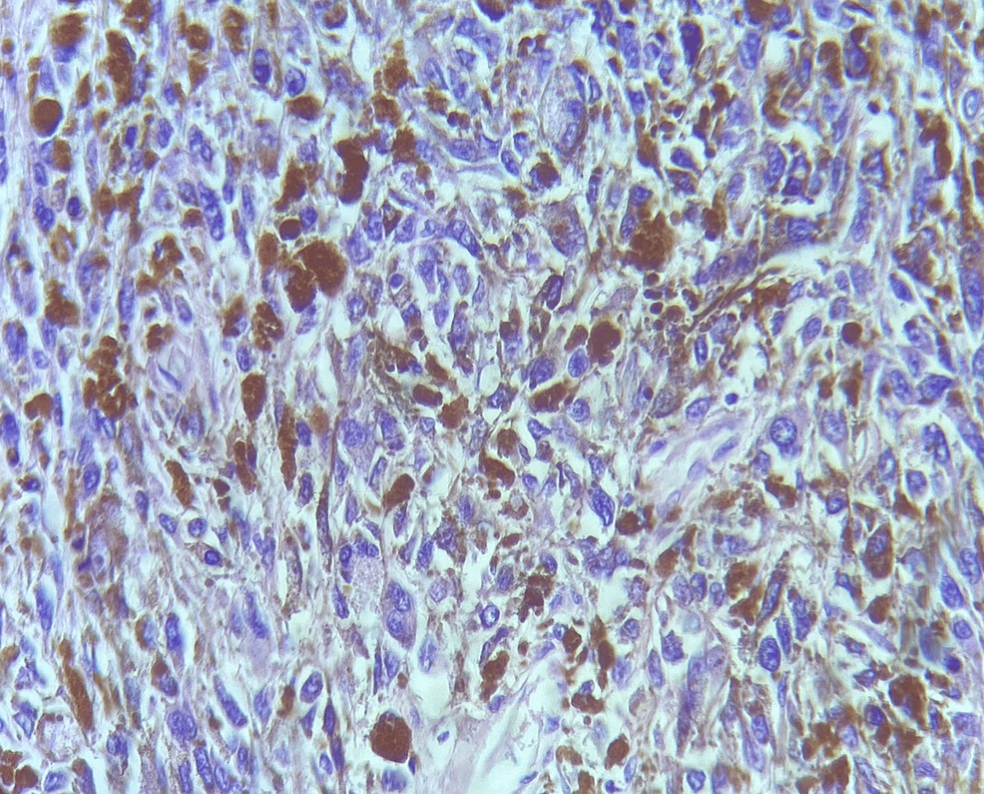
FNAB of the neck lymph node. Irregular nests of cells can be noted infiltrating the lymphoid tissue and constituting melanoma metastases. FNAB: fine needle aspiration biopsy

Furthermore, a fine needle aspiration biopsy (FNAB) of the cervical lymph node was carried out, which was positive for neoplastic cells, compatible with melanoma metastasis (Figure 4).

**Figure 5:**
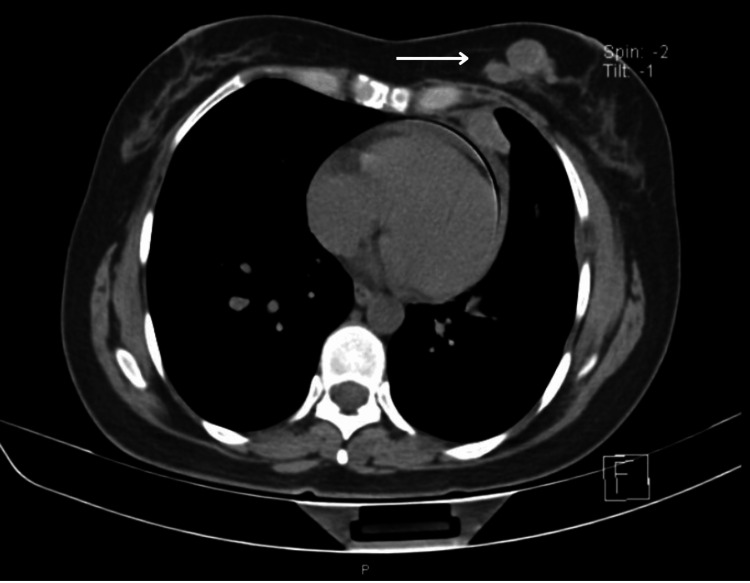
Lesion occupying the soft tissues at the level of the left breast.

**Figure 6:**
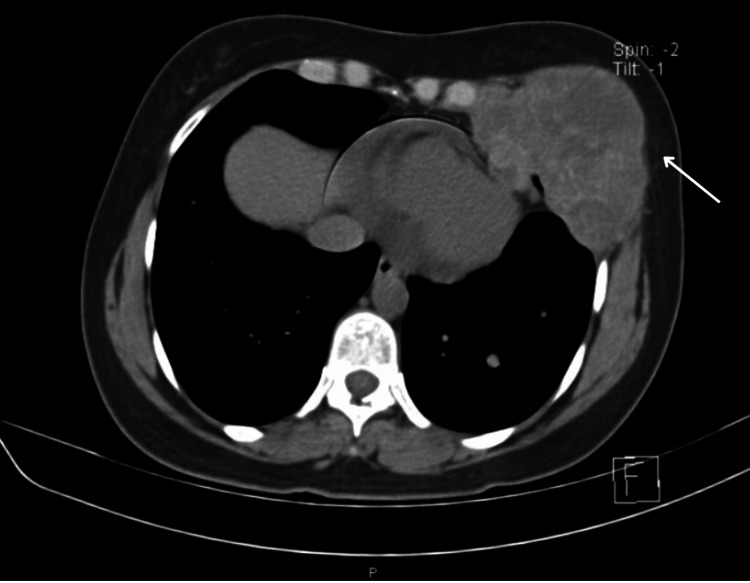
Neoplasm involving the deep fatty and muscular planes. It was also seen to have destroyed the anterior arch of the sixth rib.

**Figure 7:**
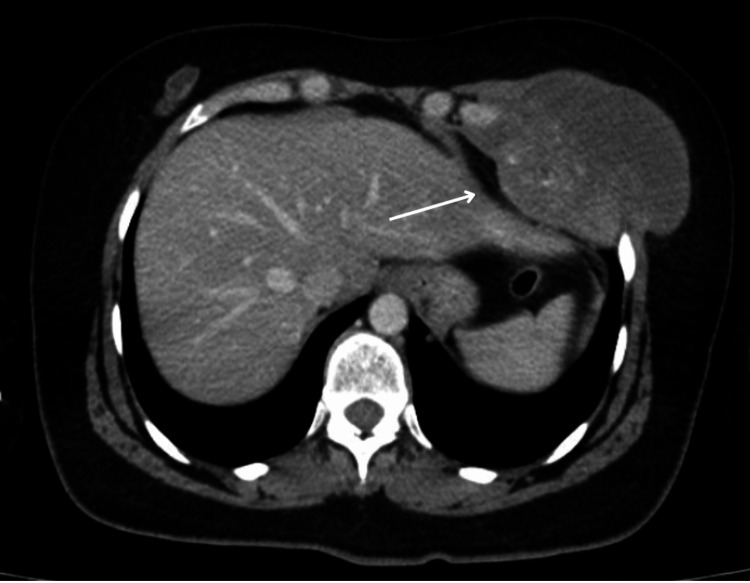
The tumor has a partial extension to the abdominal cavity.

It was decided to begin treatment with nivolumab/ipilimumab. Afterward, in control appointments, a thoracic-abdominal-pelvic CT scan was performed in a simple and contrasted phase in March 2022. The CT scan showed a lesion occupying the soft tissues at the level of the left breast and inframammary space, which involved the deep fatty and muscular planes, and also destroyed the anterior arch of the sixth rib. The lesion had an irregular morphology, lobulated borders, and isodense to the muscles, with average attenuation values ​​of 63 HU in the simple phase and 73 HU contrast-enhanced. No involvement of the superficial cutaneous planes was observed (Figures 5-6) but it presented a partial extension to the abdominal and thoracic cavities, measuring approximately 82x57 mm (Figure 7).

**Figure 8:**
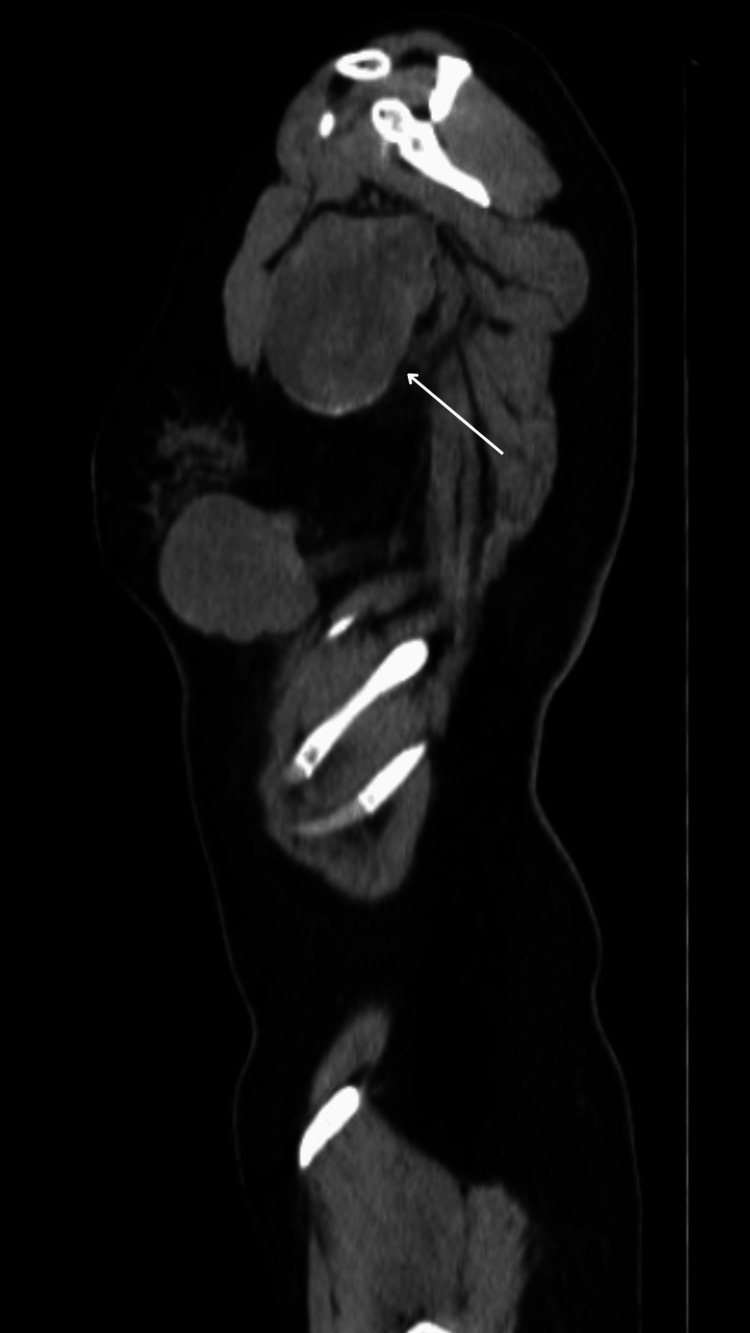
Left axillary lymph nodes with infiltrative characteristics. The largest lymph node measured 43 mm.

There was a second lesion at the level of the homolateral intercostal space, with similar characteristics, but smaller in size. It also presented intrathoracic extension with involvement of the anterior mediastinal fat. Additionally, an isodense nodule was noted in the internal quadrant of the left breast, measuring 15 mm. Left axillary lymph nodes with infiltrative characteristics, the largest of which measured 43 mm (Figure 8).

**Figure 9:**
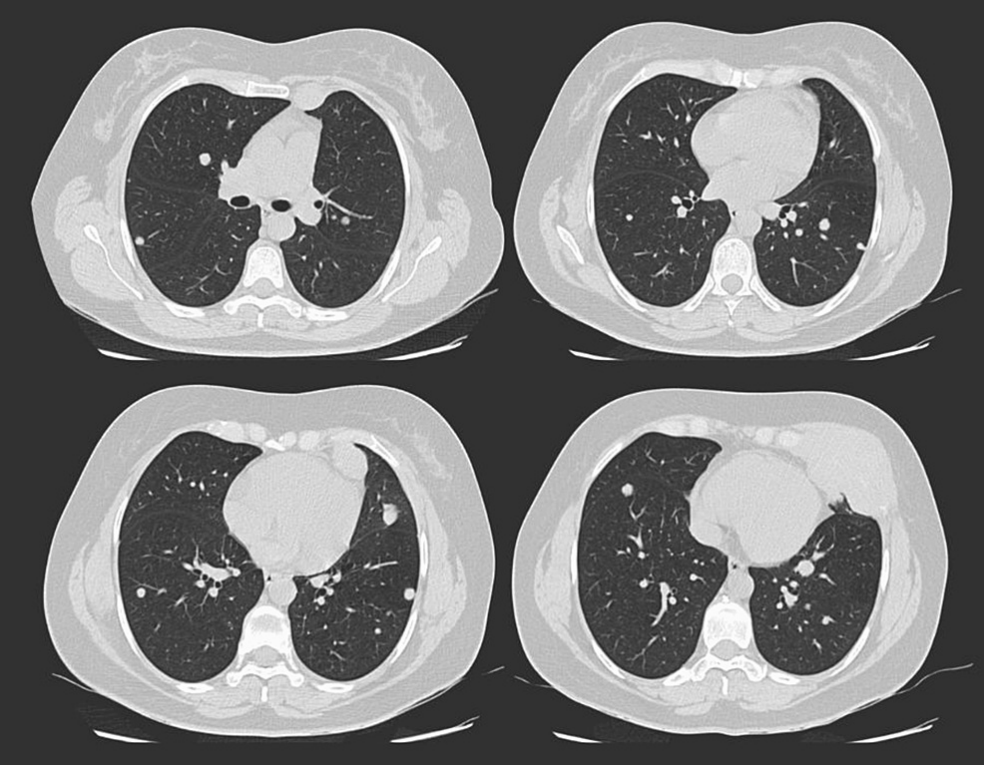
Multiple lung nodules seen in several axial slices in CT scan.

In the pulmonary window, there were multiple nodules, solid and subsolid, of diffuse and generalized distribution that involved both lungs, of various sizes (Figure 9).

**Figure 10:**
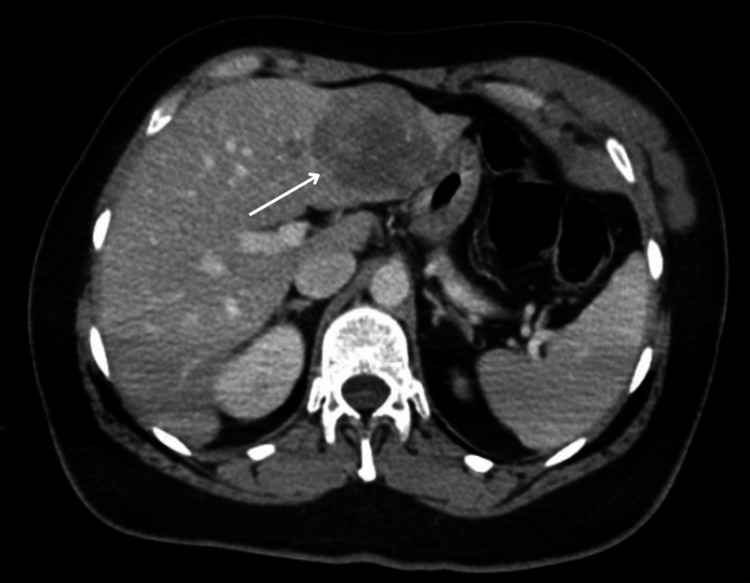
Lesion of 43 mm in the hepatic left lobe in its segments III and IV A.

CT abdomen revealed that the density of the liver parenchyma was heterogeneous, secondary to the presence of a large lesion in the left lobe in its segments III and IVA. It was predominantly hypodense but with slight peripheral contrast enhancement, maintaining internal areas with low attenuation. It measured 45 mm (Figure 10). 

The patient currently continues on treatment with immunotherapy (nivolumab/Ipilimumab) and surveillance.

## Discussion

Melanoma is one of the solid tumors with the highest number of mutations, second only to cutaneous squamous cell carcinoma [5]. It is an aggressive neoplasm of the skin and other sites such as mucosal membranes, gastrointestinal tract, uvea, and meninges [6]. The behavior of cutaneous melanoma is notoriously hostile with 90% of melanoma patients with more than three sites of metastasis succumbing to the disease within one year [7]. While primary breast cancer is vastly more common than metastasis to the breast parenchyma or subcutaneous/dermal tissue of the breast [8], it is essential to carry out a sentinel lymph node biopsy when cutaneous melanoma is greater than 0.8 mm in thickness or is ulcerated [5], as realized in our case.

The time duration for the presentation of metastases has a wide range, but the average is four years, and only a few patients present them without a previous history of melanoma [9].

Despite axillary adenopathy rarely heralding a non-mammary metastasis, metastatic melanoma should be suspected in the presence of enlarged axillary or intramammary lymph nodes, especially in cases where the primary lesion is located in the upper limb, back, or upper abdomen. The involvement of the overlying skin is uncommon; however, in rare cases, tumors with extensive lymphatic affection can present as inflammatory carcinoma. Usually, the lesion is presented as several circumscribed tumors [8,10]. It should be highlighted that sentinel lymph nodes can be located in alternative locations to the common lymphatic basins [11]. Given their rarity, the molecular characteristics of secondary (metastatic) cancers to the breast and their potential impact on therapy decisions are not well defined [12]. 

Useful histological clues to the diagnosis of breast metastasis from melanoma are intranuclear inclusions, marked nuclear pleomorphism, spindle cells, and plasmacytoid appearance; however, none of these features is specific and all can also be seen in mammary carcinoma. S100 is a very sensitive marker for melanoma and is usually diffusely positive, but it is also positive in approximately 40% of mammary carcinomas. Sox10 is also diffusely positive in most melanomas but is positive in approximately 10% of breast cancers, particularly basal-like carcinomas [13]. Melan-A and HMB45 are useful because they are more specific, but staining is sometimes focal and they are occasionally expressed by breast cancer [14].

Research is still ongoing for evidence of the overall survival benefit of a metastasectomy in melanoma [15]. For years, the standard of care for metastatic melanoma was chemotherapy, but this often failed due to the development of resistance and/or necessary dose reduction due to harsh side effects, so today it remains a historical fact [5]. Alternatively, cancer immunotherapies have demonstrated that immune system modulation can result in dramatic antitumor activity [16]. However, regarding cancer immunotherapy, approximately 20% of patients experience a long-lasting life prolongation whereas more than half of patients are still non-responders to checkpoint blockade due to specific mutations [17].

In the past, a portion of metastatic neoplasms to the breast have inevitably been misdiagnosed as breast carcinoma. In a case series study, Delair et al. reported that 12% of cases were initially diagnosed as breast carcinoma [18]. This is understood in the context of the epidemiology of breast neoplasms, so it is important to have a high clinical suspicion to regulate both the diagnostic and therapeutic approach. This scenario gives rise to a large field of research. 

## Conclusions

The diagnosis of breast metastases from cutaneous malignant melanoma is a real challenge, mainly due to its extremely rare nature, coupled with the number of genetic mutations it can present, which leads it to behave in a way that is difficult to predict and essentially aggressive. For this reason, extensive anamnesis and high clinical suspicion are crucial to determine initially this entity in order to provide the optimal treatment for the patient, despite the gloomy prognosis that this pathology represents. A diagnosis of this category requires multidisciplinary treatment and future research, despite tools currently available in terms of genomic characterization and molecular biology.
